# Death While under the Influence of Nitrous Oxide Gas

**Published:** 1888-11

**Authors:** 


DEATH WHILE UNDER THE INFLUENCE OF NITROUS OXIDE GAS.—Mrs. Elizabeth Cusick, aged sixty years, of No. 193 Third avenue, died in the dental rooms of Dr. Theodore Albright, in New York, about thirty minutes after having had some teeth extracted. Mrs. Cusick, with her daughter Mary, visited Dr. Albright for the purpose of having four teeth removed, and insisted upon taking gas, notwithstanding the dentist's advice to the contrary. She was a portly woman, and he feared she might be suffering from some form of heart trouble. He finally administered the gas, after giving her a small dose of brandy. The operation of extracting the teeth was successfully performed. Soon afterwards Mrs. Cusick complained of a feeling of oppressiveness. Medical aid was summoned and hypodermic injections were administered. Mrs. Cusick became unconscious and died soon after. Deputy Coroner Conway investigated the case and found that the woman died of fatty degeneration of the heart.

---

### Monthly Summary.

